# 3,5-Dimethyl-1-phenyl-1*H*-pyrazole-4-carbaldehyde

**DOI:** 10.1107/S1600536812010240

**Published:** 2012-03-14

**Authors:** Abdulrahman O. Al-Youbi, Abdullah M. Asiri, Hassan M. Faidallah, Seik Weng Ng, Edward R. T. Tiekink

**Affiliations:** a Chemistry Department, Faculty of Science, King Abdulaziz University, PO Box 80203, Jeddah, Saudi Arabia; b The Center of Excellence for Advanced Materials Research, King Abdulaziz University, Jeddah, PO Box 80203, Saudi Arabia; c Department of Chemistry, University of Malaya, 50603 Kuala Lumpur, Malaysia

## Abstract

In the title mol­ecule, C_12_H_12_N_2_O, the five- and six-membered rings form a dihedral angle of 68.41 (16)°. The aldehyde group is nearly coplanar with the pyrazole ring [C—C—C—O torsion angle = −0.4 (5)°]. The three-dimensional architecture is sustained by weak C—H⋯O and C—H⋯π inter­actions.

## Related literature

For the anti-bacterial properties of pyrazole derivatives, see: Kane *et al.* (2003 ▶). For related structures, see: Asiri *et al.* (2012*a* ▶,*b* ▶).
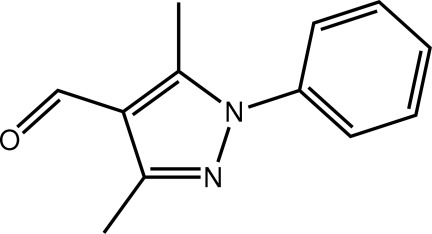


## Experimental

### 

#### Crystal data

C_12_H_12_N_2_O
*M* *_r_* = 200.24
Monoclinic, 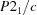

*a* = 6.6264 (4) Å
*b* = 6.7497 (4) Å
*c* = 22.6203 (12) Å
β = 94.785 (5)°
*V* = 1008.19 (10) Å^3^
*Z* = 4
Mo *K*α radiation
μ = 0.09 mm^−1^
*T* = 100 K
0.25 × 0.15 × 0.05 mm

#### Data collection

Agilent SuperNova Dual diffractometer with an Atlas detector
Absorption correction: multi-scan (*CrysAlis PRO*; Agilent, 2011 ▶) *T*_min_ = 0.979, *T*_max_ = 0.996
6376 measured reflections
2335 independent reflections
1951 reflections with *I* > 2σ(*I*)
*R*_int_ = 0.035

#### Refinement

*R*[*F* ^2^ > 2σ(*F* ^2^)] = 0.080
*wR*(*F* ^2^) = 0.194
*S* = 1.23
2335 reflections
138 parameters
H-atom parameters constrained
Δρ_max_ = 0.43 e Å^−3^
Δρ_min_ = −0.31 e Å^−3^

### 

Data collection: *CrysAlis PRO* (Agilent, 2011 ▶); cell refinement: *CrysAlis PRO*; data reduction: *CrysAlis PRO*; program(s) used to solve structure: *SHELXS97* (Sheldrick, 2008 ▶); program(s) used to refine structure: *SHELXL97* (Sheldrick, 2008 ▶); molecular graphics: *ORTEP-3* (Farrugia, 1997 ▶) and *DIAMOND* (Brandenburg, 2006 ▶); software used to prepare material for publication: *publCIF* (Westrip, 2010 ▶).

## Supplementary Material

Crystal structure: contains datablock(s) global, I. DOI: 10.1107/S1600536812010240/xu5480sup1.cif

Structure factors: contains datablock(s) I. DOI: 10.1107/S1600536812010240/xu5480Isup2.hkl

Supplementary material file. DOI: 10.1107/S1600536812010240/xu5480Isup3.cml

Additional supplementary materials:  crystallographic information; 3D view; checkCIF report

## Figures and Tables

**Table 1:** Hydrogen-bond geometry (Å, °) *Cg*1 is the centroid of the C7–C12 ring.

*D*—H⋯*A*	*D*—H	H⋯*A*	*D*⋯*A*	*D*—H⋯*A*
C8—H8⋯O1^i^	0.95	2.43	3.315 (4)	155
C11—H11⋯*Cg*1^ii^	0.95	2.71	3.509 (4)	142

Symmetry codes: (i) 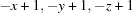; (ii) 
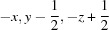
.

**Fig. 1.**
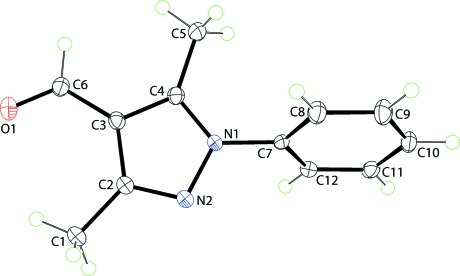
The molecular structure of (I) showing the atom-labelling scheme and displacement ellipsoids at the 50% probability level.

**Fig. 2.**
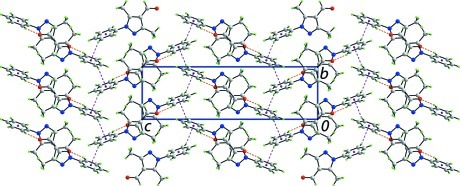
A view in projection down the a axis of the unit-cell contents of (I). The C—H···O and C—H···π interactions are shown as orange and purple dashed lines, respectively.

## supplementary crystallographic information

### Comment

In continuation of structural studies of pyrazole derivatives (Asiri *et al.*, 2012*a*; Asiri *et al.*, 2012*b*), motivated by their putative biological activity (Kane *et al.*, 2003), the title compound, 3,5-dimethyl-1-phenyl-1*H*-4-pyrazole-3-carboxaldehyde (I), was investigated crystallographically.

In (I), Fig. 1, there is a twist about the single bond linking the five- and six-membered rings with the N2—N1—C7—C8 torsion angle being -112.1 (3) °; the dihedral angle between the rings is 68.41 (16) °. The aldehyde group is co-planar with the pyrazole ring to which it is connected as seen in the value of the C2—C3—C6—O1 torsion angle of -0.4 (5)°.

Molecules are connected into the three-dimensional architecture by C—H···O and C—H···π interactions, Fig. 2 and Table 1.

### Experimental

To a cold solution of N,N-dimethylformamide (1.46 g, 20 mmol), freshly distilled phosphorous oxychloride (1.54 g, 10 mmol) was added with stirring over a period of 30 min. A solution of 3,5-dimethyl-1-phenyl-1*H*-4-pyrazole-3-carboxaldehyde (1.72 g, 10 mmol) in N,N-dimethylformamide (15 ml) was added drop-wise while maintaining the temperature between 273–278 K. The resulting mixture was heated under reflux for 1 h, cooled and poured with continuous stirring into crushed ice. After 15 min, the precipitate was filtered and crystallized from aqueous ethanol to give needles. Yield: 69%. *M*.pt: 397–399 K.

### Refinement

Carbon-bound H-atoms were placed in calculated positions [C—H = 0.95 to 0.98 Å, *U*_iso_(H) = 1.2 to 1.5*U*_eq_(C)] and were included in the refinement in the riding model approximation. Owing to poor agreement, the (2 1 0) reflection was omitted from the final cycles of refinement.

### Crystal data

**Table:**

C_12_H_12_N_2_O	*F*(000) = 424
*M**_r_* = 200.24	*D*_x_ = 1.319 Mg m^−^^3^
Monoclinic, *P*2_1_/*c*	Mo *K*α radiation, λ = 0.71073 Å
Hall symbol: -P 2ybc	Cell parameters from 2660 reflections
*a* = 6.6264 (4) Å	θ = 2.7–27.5°
*b* = 6.7497 (4) Å	µ = 0.09 mm^−^^1^
*c* = 22.6203 (12) Å	*T* = 100 K
β = 94.785 (5)°	Prism, colourless
*V* = 1008.19 (10) Å^3^	0.25 × 0.15 × 0.05 mm
*Z* = 4	

### Data collection

**Table:**

Agilent SuperNova Dual diffractometer with an Atlas detector	2335 independent reflections
Radiation source: SuperNova (Mo) X-ray Source	1951 reflections with *I* > 2σ(*I*)
Mirror monochromator	*R*_int_ = 0.035
Detector resolution: 10.4041 pixels mm^-1^	θ_max_ = 27.6°, θ_min_ = 3.1°
ω scan	*h* = −8→8
Absorption correction: multi-scan (*CrysAlis PRO*; Agilent, 2011)	*k* = −8→6
*T*_min_ = 0.979, *T*_max_ = 0.996	*l* = −29→29
6376 measured reflections	

### Refinement

**Table:**

Refinement on *F*^2^	Primary atom site location: structure-invariant direct methods
Least-squares matrix: full	Secondary atom site location: difference Fourier map
*R*[*F*^2^ > 2σ(*F*^2^)] = 0.080	Hydrogen site location: inferred from neighbouring sites
*wR*(*F*^2^) = 0.194	H-atom parameters constrained
*S* = 1.23	*w* = 1/[σ^2^(*F*_o_^2^) + (0.0317*P*)^2^ + 2.9806*P*] where *P* = (*F*_o_^2^ + 2*F*_c_^2^)/3
2335 reflections	(Δ/σ)_max_ = 0.001
138 parameters	Δρ_max_ = 0.43 e Å^−^^3^
0 restraints	Δρ_min_ = −0.31 e Å^−^^3^

### Fractional atomic coordinates and isotropic or equivalent isotropic displacement parameters (Å^2^)

**Table:**

	*x*	*y*	*z*	*U*_iso_*/*U*_eq_	
O1	0.2480 (3)	0.6126 (4)	0.58330 (9)	0.0215 (5)	
N1	0.2395 (4)	0.2683 (4)	0.40971 (10)	0.0148 (5)	
N2	0.2307 (4)	0.1335 (4)	0.45524 (11)	0.0195 (6)	
C1	0.2332 (6)	0.1451 (5)	0.56361 (14)	0.0276 (8)	
H1A	0.1190	0.0526	0.5626	0.041*	
H1B	0.2177	0.2463	0.5940	0.041*	
H1C	0.3600	0.0727	0.5730	0.041*	
C2	0.2378 (5)	0.2431 (5)	0.50395 (13)	0.0172 (6)	
C3	0.2500 (4)	0.4477 (5)	0.49014 (13)	0.0148 (6)	
C4	0.2499 (4)	0.4563 (5)	0.42869 (13)	0.0153 (6)	
C5	0.2611 (6)	0.6283 (5)	0.38750 (14)	0.0236 (7)	
H5A	0.1524	0.6179	0.3555	0.035*	
H5B	0.3925	0.6280	0.3706	0.035*	
H5C	0.2456	0.7519	0.4094	0.035*	
C6	0.2550 (4)	0.6179 (5)	0.52932 (13)	0.0167 (6)	
H6	0.2644	0.7448	0.5116	0.020*	
C7	0.2304 (4)	0.1953 (4)	0.34975 (12)	0.0143 (6)	
C8	0.4000 (5)	0.2103 (5)	0.31798 (14)	0.0208 (7)	
H8	0.5214	0.2683	0.3354	0.025*	
C9	0.3893 (5)	0.1389 (5)	0.25999 (14)	0.0235 (7)	
H9	0.5033	0.1502	0.2374	0.028*	
C10	0.2120 (5)	0.0509 (5)	0.23500 (13)	0.0203 (7)	
H10	0.2060	0.0009	0.1956	0.024*	
C11	0.0447 (5)	0.0363 (5)	0.26753 (13)	0.0182 (6)	
H11	−0.0756	−0.0246	0.2505	0.022*	
C12	0.0520 (5)	0.1106 (4)	0.32508 (13)	0.0165 (6)	
H12	−0.0636	0.1034	0.3472	0.020*	

### Atomic displacement parameters (Å^2^)

**Table:**

	*U*^11^	*U*^22^	*U*^33^	*U*^12^	*U*^13^	*U*^23^
O1	0.0239 (11)	0.0255 (12)	0.0150 (10)	−0.0002 (10)	0.0007 (8)	−0.0037 (9)
N1	0.0210 (12)	0.0131 (12)	0.0105 (11)	−0.0012 (10)	0.0024 (9)	0.0006 (9)
N2	0.0305 (14)	0.0157 (13)	0.0126 (12)	−0.0021 (11)	0.0031 (10)	0.0009 (10)
C1	0.048 (2)	0.0210 (17)	0.0140 (15)	−0.0048 (16)	0.0052 (14)	0.0023 (13)
C2	0.0219 (15)	0.0166 (14)	0.0134 (14)	−0.0009 (12)	0.0020 (11)	0.0008 (11)
C3	0.0144 (13)	0.0171 (14)	0.0133 (13)	−0.0012 (12)	0.0031 (10)	−0.0005 (11)
C4	0.0157 (13)	0.0157 (15)	0.0140 (14)	−0.0013 (12)	−0.0020 (11)	0.0000 (11)
C5	0.0401 (19)	0.0145 (15)	0.0160 (15)	−0.0003 (15)	0.0016 (13)	−0.0004 (12)
C6	0.0170 (14)	0.0173 (15)	0.0157 (14)	−0.0012 (12)	0.0012 (11)	−0.0024 (12)
C7	0.0218 (14)	0.0092 (13)	0.0118 (13)	0.0006 (11)	0.0004 (11)	0.0001 (10)
C8	0.0211 (15)	0.0238 (16)	0.0175 (15)	−0.0033 (13)	0.0017 (12)	−0.0043 (13)
C9	0.0230 (15)	0.0280 (18)	0.0205 (15)	−0.0003 (14)	0.0075 (12)	−0.0071 (14)
C10	0.0298 (17)	0.0183 (15)	0.0129 (14)	0.0024 (13)	0.0012 (12)	−0.0031 (12)
C11	0.0242 (15)	0.0139 (14)	0.0155 (14)	−0.0013 (12)	−0.0038 (11)	−0.0001 (12)
C12	0.0206 (14)	0.0140 (14)	0.0149 (13)	−0.0016 (12)	0.0024 (11)	0.0008 (11)

### Geometric parameters (Å, º)

**Table:**

O1—C6	1.226 (4)	C5—H5B	0.9800
N1—C4	1.339 (4)	C5—H5C	0.9800
N1—N2	1.379 (3)	C6—H6	0.9500
N1—C7	1.439 (4)	C7—C12	1.387 (4)
N2—C2	1.325 (4)	C7—C8	1.388 (4)
C1—C2	1.506 (4)	C8—C9	1.394 (4)
C1—H1A	0.9800	C8—H8	0.9500
C1—H1B	0.9800	C9—C10	1.393 (5)
C1—H1C	0.9800	C9—H9	0.9500
C2—C3	1.419 (4)	C10—C11	1.385 (4)
C3—C4	1.391 (4)	C10—H10	0.9500
C3—C6	1.450 (4)	C11—C12	1.392 (4)
C4—C5	1.494 (4)	C11—H11	0.9500
C5—H5A	0.9800	C12—H12	0.9500
			
C4—N1—N2	112.9 (2)	H5A—C5—H5C	109.5
C4—N1—C7	128.6 (2)	H5B—C5—H5C	109.5
N2—N1—C7	118.5 (2)	O1—C6—C3	125.8 (3)
C2—N2—N1	104.6 (2)	O1—C6—H6	117.1
C2—C1—H1A	109.5	C3—C6—H6	117.1
C2—C1—H1B	109.5	C12—C7—C8	121.4 (3)
H1A—C1—H1B	109.5	C12—C7—N1	119.2 (3)
C2—C1—H1C	109.5	C8—C7—N1	119.4 (3)
H1A—C1—H1C	109.5	C7—C8—C9	118.8 (3)
H1B—C1—H1C	109.5	C7—C8—H8	120.6
N2—C2—C3	111.0 (3)	C9—C8—H8	120.6
N2—C2—C1	119.9 (3)	C10—C9—C8	120.3 (3)
C3—C2—C1	129.1 (3)	C10—C9—H9	119.9
C4—C3—C2	105.4 (3)	C8—C9—H9	119.9
C4—C3—C6	125.2 (3)	C11—C10—C9	120.0 (3)
C2—C3—C6	129.4 (3)	C11—C10—H10	120.0
N1—C4—C3	106.1 (3)	C9—C10—H10	120.0
N1—C4—C5	122.7 (3)	C10—C11—C12	120.3 (3)
C3—C4—C5	131.3 (3)	C10—C11—H11	119.8
C4—C5—H5A	109.5	C12—C11—H11	119.8
C4—C5—H5B	109.5	C7—C12—C11	119.1 (3)
H5A—C5—H5B	109.5	C7—C12—H12	120.4
C4—C5—H5C	109.5	C11—C12—H12	120.4
			
C4—N1—N2—C2	−0.6 (3)	C6—C3—C4—C5	2.2 (5)
C7—N1—N2—C2	−178.7 (3)	C4—C3—C6—O1	177.3 (3)
N1—N2—C2—C3	0.3 (3)	C2—C3—C6—O1	−0.4 (5)
N1—N2—C2—C1	−179.5 (3)	C4—N1—C7—C12	−110.1 (4)
N2—C2—C3—C4	0.0 (4)	N2—N1—C7—C12	67.7 (4)
C1—C2—C3—C4	179.8 (3)	C4—N1—C7—C8	70.2 (4)
N2—C2—C3—C6	178.1 (3)	N2—N1—C7—C8	−112.1 (3)
C1—C2—C3—C6	−2.2 (6)	C12—C7—C8—C9	0.2 (5)
N2—N1—C4—C3	0.6 (3)	N1—C7—C8—C9	179.9 (3)
C7—N1—C4—C3	178.5 (3)	C7—C8—C9—C10	−1.1 (5)
N2—N1—C4—C5	179.9 (3)	C8—C9—C10—C11	0.8 (5)
C7—N1—C4—C5	−2.2 (5)	C9—C10—C11—C12	0.5 (5)
C2—C3—C4—N1	−0.3 (3)	C8—C7—C12—C11	1.1 (5)
C6—C3—C4—N1	−178.5 (3)	N1—C7—C12—C11	−178.7 (3)
C2—C3—C4—C5	−179.6 (3)	C10—C11—C12—C7	−1.4 (5)

### Hydrogen-bond geometry (Å, º)

Cg1 is the centroid of the C7–C12 ring.

**Table:**

*D*—H···*A*	*D*—H	H···*A*	*D*···*A*	*D*—H···*A*
C8—H8···O1^i^	0.95	2.43	3.315 (4)	155
C11—H11···*Cg*1^ii^	0.95	2.71	3.509 (4)	142

Symmetry codes: (i) −*x*+1, −*y*+1, −*z*+1; (ii) −*x*, *y*−1/2, −*z*+1/2.
